# Role of tight junction-associated MARVEL protein marvelD3 in migration and epithelial–mesenchymal transition of hepatocellular carcinoma

**DOI:** 10.1080/19336918.2021.1958441

**Published:** 2021-08-02

**Authors:** Yanmeng Li, Teng Li, Donghu Zhou, Jia Wei, Zhenkun Li, Xiaojin Li, Siyu Jia, Qin Ouyang, Saiping Qi, Zhibin Chen, Bei Zhang, Jing Yu, Jidong Jia, Anjian Xu, Jian Huang

**Affiliations:** a Experimental Center, Beijing Friendship Hospital, Capital Medical University, Beijing, China; b Liver Research Center, Beijing Friendship Hospital, Capital Medical University, Beijing, China; c National Clinical Research Center for Digestive Disease, Beijing Friendship Hospital, Capital Medical University, Beijing, China; d Department of Medical Oncology, National Cancer Center/National Clinical Research Center for Cancer/Cancer Hospital, Chinese Academy of Medical Sciences and Peking Union Medical College, Beijing, People’s Republic of China; e Department of Oncology, Beijing Friendship Hospital, Capital Medical University, Beijing, China

**Keywords:** Hepatocellular carcinoma, marvelD3, EMT, migration, NF-κB

## Abstract

MarvelD3, a recently identified tight junction membrane protein, could be associated with hepatocellular carcinoma (HCC). We aimed to investigate the role of marvelD3 in Epithelial–Mesenchymal Transition (EMT) and migration of HCC and explore the underlying molecular mechanisms. First, we assessed marvlD3 expression in HCC and normal liver tissues and found loss of marvelD3 expression was significantly correlated with the occurrence and TNM stage of HCC. Second, we detected that marvelD3 was downregulated in HCC cells with transforming growth factor β1 and snail/slug-induced EMT. Finally, we analyzed expression of marvelD3 protein was significantly associated with EMT and the NF-κB signaling pathway. Our study demonstrated that MarvelD3 inhibited EMT and migration of HCC cells along with inhibiting NF-κB signaling pathway.

**Abbreviations:** **HCC**, Hepatocellular carcinoma; **TJ**, Tight junction; **MARVEL**, MAL and related proteins for vesicle trafficking and membrane link; **EMT**, Epithelial**–**mesenchymal transition; **NF-κB**, Nuclear factor kappa B; **TAMPs**, Tight junction-associated marvel proteins; **TGF-β1**, Transforming growth factor-β1; **MMP9**, matrix metallopeptidase 9; **RT-PCR**, Real-time PCR; **IHC**, Immunohistochemistry; **IF**, Immunofluorescence.

## Introduction

Tight junctions (TJs), which are an intercellular adhesion complex of epithelial and endothelial cells, are very important for the growth of metastatic cancer cells. TJs separate the internal space of multicellular organisms from external compartments and form a diffusion barrier, which allows regulated movement of ions and solutes through the paracellular pathway [1,2]. TJs consist of tight junction-associated marvel proteins (TAMPs) (occludin, tricellulin, and marvelD3) and claudins. Occludin, marvelD3 and claudin proteins are the major integral components of bicellular TJs [2,3]. Claudins regulate the interactions between occludin, tricellulin, and marvelD3, which inversely modulate claudin oligomerization [2]. Their cross-linking functions have been associated with the regulation of signal transduction mechanisms that lead to epithelial cell proliferation and differentiation [4]. Moreover, deregulation of TJ expression has been reported in cancers [5–9]. TJ proteins are emerging as targets for novel therapeutic approaches for liver disease. Recent studies demonstrate that TJ protein expression is altered in hepatocellular carcinoma (HCC) [10]. For example, claudin-1 has been shown to be up-regulated in advanced liver disease and HCC [11].

MarvelD3 was recently discovered as a TAMP member. It has two isoforms and shows a broad tissue distribution. Knockdown of marvelD3 affects the paracellular barrier properties of TJs [1]. MarvelD3 functions as a regulator of epithelial cell proliferation, migration, and survival in human colon and pancreatic cancer cells [6,12]. However, the pathological significance of MarvelD3 is unclear in HCC.

Owing to the intrahepatic and extrahepatic metastases, HCC is characterized by high recurrence and low 5-y survival rates [13]. It has been demonstrated that epithelial**–**mesenchymal transition (EMT) plays a pivotal role in the early events of HCC [14]. There is accumulating evidence of a major link between EMT and tumor cell metastasis for strong motility and invasiveness [15]. Among all known signaling pathways involved in EMT, the transforming growth factor-β1 (TGF-β1) and snail-induced signaling pathways have been recognized to be responsible for the initiation, progression, and metastasis of HCC [16–19]. Claudin-3 inhibits EMT and invasion of lung squamous cell carcinoma cell [20]. Downregulation of marvelD3 is involved in EMT of human pancreatic cancer cells [6]. However, it is uncertain whether marvelD3 is also involved in the EMT process in HCC.

Additionally, some pathways, such as nuclear factor kappa B (NF-κB) and mitogen-activated protein kinase 1 pathways, have been investigated in EMT and metastasis of HCC, which perform biological functions through the interactions with a variety of cellular factors, including TGF-β [21–23]. Some inhibitors regulate TJ-related proteins and suppress invasion and metastasis through NF- κB/Snail inhibition [24,25]. The association between MarvelD3 with NF-κB pathway is worth exploring.

Previous studies have demonstrated that the correlation between marvelD3 and other cancers [12]. However, the novel role of marvelD3 in HCC remains unclear. In this study, we aimed to clarify the regulatory mechanisms of marvelD3 in HCC development, which supplements current understanding of HCC progression and provides potential prognostic and therapeutic targets for HCC treatment.

## Materials and methods

### Cell culture and human liver sample

Human liver cancer cell line Hep3B was cultured in RPMI 1640 (Sigma, USA) supplemented with 10% fetal bovine serum (FBS, Sigma), 100 U/mL penicillin, and 100 U/mL streptomycin at 37°C with 5% CO_2_. Huh-7 cells were cultured in Dulbecco’s modification of Eagle’s medium (Sigma) with 10% FBS. Huh-7 cell line is a P53 mutation cell line and Hep3B is a P53 null cell line. Cells in the exponential growth phase were used in experiments.

All experiments involving human tissues were evaluated by the local ethics committee (Beijing You-an Hospital, Capital Medical University, Beijing, China, EC-B-031-A02-9.0). The sample was collected from a subject who provided informed consent for their tissue to be used for research purposes. For immunochemistry, normal liver tissue (n = 13) and liver tumor tissue (n = 25) were obtained as paraffin blocks and sectioned at 4 µm thicknesses. Normal and tumor tissues were evaluated by two pathologists. Tissues for Western blotting (n = 4) and RT-PCR (n = 3), which included liver cancer (C) and paired normal liver tissues (N), were frozen tissue samples. The patient characteristics are listed in Table S1.

### Antibodies and reagents

An anti-marvelD3 antibody (ab118916), anti-Claudin 3 antibody (ab15102) and anti-GAPDH (ab6276) were purchased from Abcam (USA). An anti-E-cadherin antibody (#3195) and anti-vimentin antibody (#5741) were purchased from Cell Signaling Technology (MA, USA). An anti-NF-kB antibody kit (#9936) was purchased from Cell Signaling Technology. Horseradish peroxidase (HRP)-conjugated goat anti-mouse and goat anti-rabbit IgGs were purchased from Zhongshan Jinqiao (Beijing China). TGF-β1 (PeproTech, USA) was dissolved in citric acid (pH 3.0) at a concentration of 10 µg/mL, stored at −20°C, and diluted in culture medium to the required concentration of 10 ng/mL. NF-κB inhibitor BAY 11–7082 (HY-13,453; MCE) were added to the culture medium at a final concentration of 20 µM in accordance with the manufacturer’s instructions.

### siRNA-mediated transient knockdown of marvelD3 expression

MarvelD3 knockdown was performed using siRNA ID stB0014718A (RiboBio, Guangzhou, China) to target the marvelD3-coding region, and the negative siRNA (ID siN0000001-1-5) was used as contrast. In totL, 5 × 10^5^ Huh-7 and Hep3B cells were transfected with 20 nM siRNA using LTX reagent (Invitrogen, USA) in accordance with the manufacturer’s instructions. The culture medium was replaced after 12 hours and subsequent assays were performed after 24 or 48 hours.

### Plasmid transfection and marvelD3 expression

The entire coding sequences of human snail and slug were inserted into the mammalian expression plasmid pcDNA3.1. The marvelD3 fragment was cloned into the PCDH-CMV-MCS-EF1-cop GFP-T2A-Puro plasmid vector between EcoR1 and Not1 sites. HCC cells were transfected with the constructed plasmid using transfection reagent LTX (Invitrogen, USA) in accordance with the manufacturer’s instructions. The culture medium was changed at 6 hours after transfection and the cells were harvested at 24 or 48 hours after transfection for subsequent assays.

### Cell survival assay and transwell migration assay

The effect of marvelD3 on HCC cells viability was determined by MTS cell proliferation assay kit (Promega, Madison, WI, USA) in accordance with the manufacturer’s instructions. HCC cells (5 × 10^3^ cells/well) were seeded into a 96‐well plate. The OD value was read after 24 and 48 h. For the migration assay, we used modified Boyden chambers with filter inserts (8 μm pore size). Approximately 1 × 10^5^ cells in 200 μL of medium were placed in the upper chamber, and 1 ml of complete medium was placed in the lower chamber. After 24**–**48 h in culture, cells were fixed in 4% paraformaldehyde for 30 min and then stained with 0.05% crystal violet in deionized water for 2 h. Cells on the upper side of the filters were removed with cotton-tipped swabs and the filters were washed with PBS. Cells on the underside of the filters were viewed and counted under a microscope.

### Wound healing assay

HCC cells were seeded into a six-well plate and cultured overnight. The next day, after reaching approximately ≥90% confluence, three linear wounds were created using a 200 µl pipette tip. Cells were washed with PBS and cultured for 24 h. Photographs of the wounded area were obtained immediately after making the scratch (0 h time point) and at 24 h to monitor the wounding healing capacity of the cells. The experiment was repeated at least once.

### Real-time PCR (RT-PCR)

Total RNA was extracted from HCC cells and liver tissues using TRI Reagent (Sigma) in accordance with the manufacturer’s instructions. A total of 2 μg RNA was used for cDNA synthesis with a Reverse Transcription Kit (Roche, Germany) in a 20 μl reaction. Equal amounts of cDNA were subjected to PCR using the following conditions: initial denaturation at 95°C for 10 min, followed by 40 cycles of 95°C for 15 s and 60°C for 1 min, and terminal extension at 72°C for 5 min. Each sample was examined in triplicate. *GAPDH* was used as the internal control. Real-time PCR primers are listed in supporting Table S2. GraphPad Prism v7 (GraphPad Software, San Diego, CA, USA) was used to create histograms.

### Western blotting

Proteins were extracted from HCC cells and fresh liver tissue by lysis in RIPA buffer with protease and phosphatase inhibitors (Roche, USA). Equal quantities of proteins were separated by 12% SDS-PAGE and transferred onto a PVDF membrane (GE, USA) using a Bio-Rad wet transfer unit. After blocking with 5% (w/v) nonfat dried milk in TBST [25 mM Tris, pH 7.5, 150 mM NaCl, and 0.1% (v/v) Tween-20] for 1 h at room temperature, the membrane was incubated with a primary antibody overnight at 4°C, followed by HRP-conjugated goat anti-mouse or goat anti-rabbit IgGs (1:5000) for 1 h at room temperature. Immunocomplexes on the membrane were visualized with Immobilon Western Chemiluminescent HRP Substrate (Millipore USA) using Image Lab Software (BIO-RAD).

### Immunofluorescence (IF)

Treated HCC cells were washed three times in PBS, fixed for 30 min in 4% paraformaldehyde in PBS, and permeabilized for 10 min with 0.3% Triton X-100 in PBS. Nonspecific binding sites were blocked by 60 min of incubation in 5% bovine serum albumin in PBS. Then, the cells were incubated with primary antibody against anti-E-cadherin or anti-vimentin (1:200) at 4°C overnight. After three 5-min washes with PBS, the cells were incubated with a mixture of anti-rabbit Alexa fluor 488-conjugated secondary antibodies (1:200) for 2 h at room temperature. Following three 5-min wash with PBS, the cells were mounted on a slide in mounting medium with DAPI (Molecular Probe, USA). Cells were examined and photographed under a confocal microscope (FV 300, Olympus).

### Immunohistochemistry (IHC)

Liver tissues were fixed in 10% neutral buffered formalin, embedded in paraffin, and stained for routine histology. Sections were dewaxed in xylene and dehydrated in alcohol. Antigen retrieval was performed by microwaving in citric saline for 15 min. Thin sections were deparaffinized and treated with 0.3% hydrogen peroxide for 15 min to block endogenous peroxidase activity. The sections were blocked further in 3% bovine serum albumin, followed by incubation with a primary antibody against E-cadherin (1:400) or marvelD3 (1:500) for 20 h at 4°C. After rinsing, the sections were incubated with a biotinylated secondary antibody for 60 min at room temperature. Protein expression was visualized by 3,3ʹ-diaminobenzidine tetrahydrochloride staining. The sections were counterstained with hematoxylin before dehydration for 30 s. Staining intensities were determined by measuring the integrated optical density (IOD) by light microscopy using Image-Pro v6.0.

### Statistical analysis

All experiments were carried out at least three times. Data are represented as means ± SEM. Differences between groups were calculated using the Student’s t-test. Statistical significance was defined as P < 0.05 for a two-tailed test. All statistical analyses were carried out with Graphpad Prism 7.0 software.

## Results

### Associations of reduced marvelD3 expression with altered EMT markers in HCC tissues compared with normal liver tissues

**Figure 1.**
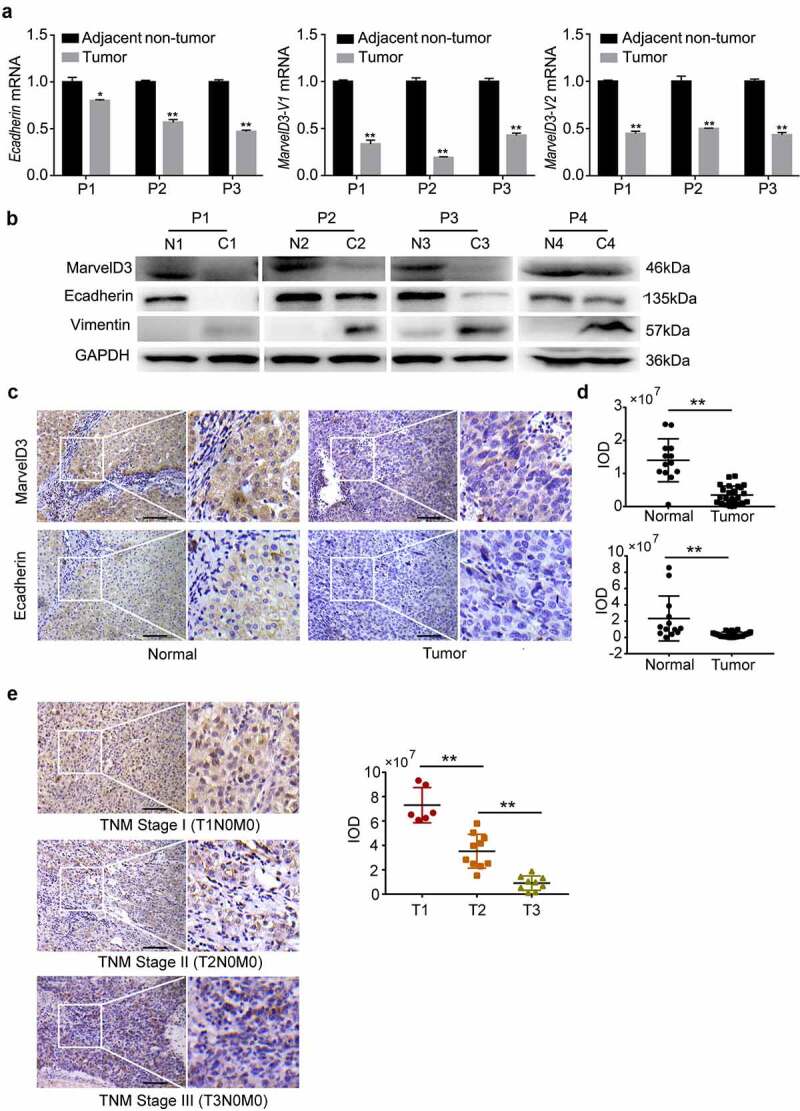
Association of reduced marvelD3 expression with altered EMT markers in HCC tissues compared with adjacent normal tissues

In this study, we first confirmed differential marvelD3 expression in HCC tissues vs. normal liver tissue. Western blot and RT-PCR assays revealed reduced marvelD3 expression in HCC tissue compared with paired adjacent normal tissues and a decrease of E-cadherin and increase of vimentin (Figure 1(a,b)). IHC data were consistent with the western blot data. E-cadherin and marvelD3 protein levels were significantly lower in HCC tissues regardless of low or high grades compared with normal tissues (Figure 1(c,d)), and lower expression of marvelD3 was observed in high TNM stage HCC tissue than in low grade (Figure 1(e)).

**(a)** The expression of E-cadherin and MarvelD3 mRNA was determined by RT-PCR in three pairs of HCC tissues; **(b)** The expression of E-cadherin, Vimentin and MarvelD3 protein were analyzed by western blotting in four pairs of HCC tissues; (N: normal liver tissues; C: HCC tissues); **(c)** Representative IHC images of E-cadherin and MarvelD3 staining in HCC tumor or normal liver tissues (scale bar, 100 μm); **(d)** IHC scores of normal liver tissue (*n* = 13) and liver tumor tissue (*n* = 25) on E-cadherin and MarvelD3 staining; **(e)** MarvelD3 IHC staining and score in different TNM stage HCC tumor were analyzed.

### Downregulation of marvelD3 during TGF-β1 and snail-induced EMT in HCC cells

**Figure 2.**
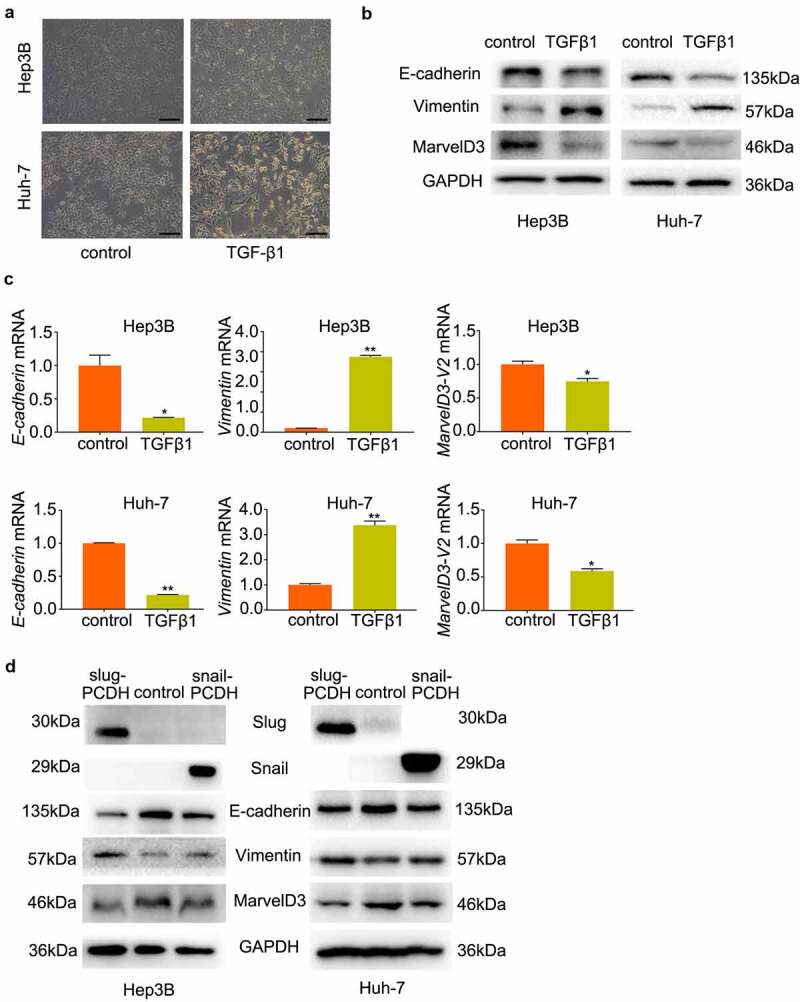
Downregulation of marvelD3 during TGF-β1 and snail-induced EMT in HCC cells

To detect the function of marvelD3 in EMT of HCC cells, HCC cells (Hep3B and Huh-7) were stimulated with TGF-β1 (10 ng/mL) for 48 h. Results showed that the TGF-β1 treated cells exhibited remarkable morphological changes into spindle-shaped cells with loose cell connections (Figure 2(a)). As well as reduced mRNA and protein expression of E-cadherin and marvelD3, expression of vimentin was upregulated (Figure 2(b,c)). During snail and slug-induced EMT of HCC cells, E-cadherin and marvelD3 proteins were decreased, while the vimentin protein level was increased (Figure 2(d)).

**(a)** HCC cells stimulated with TGF-β1 exhibited morphological changes into spindle-shaped cells with loose cell connections. Observations were made using a light microscope (×100). **(b) and (c)** The expression of E-cadherin, Vimentin, and MarvelD3 was tested in HCC cells treated with TGF-β1 by western blot (b) and RT-PCR (c). **(d)** The expression of E-cadherin, Vimentin, and MarvelD3 was evaluated in snail- and slug-transfected HCC cells by western blot. (*P < 0.05, **P < 0.01)

### Effects of marvelD3 on regulation of EMT gene expression in vitro

**Figure 3.**
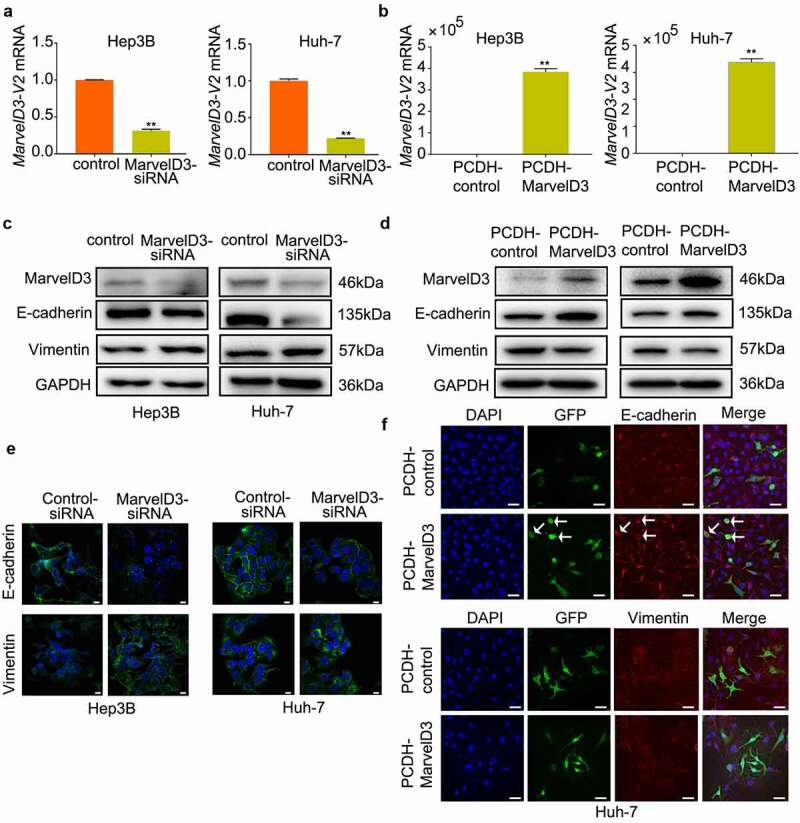
Effects of marvelD3 on regulation of EMT gene expression in vitro

Many studies have revealed that EMT increases the incidence of cancer metastasis [26,27]. Therefore, we ascertained whether marvelD3 reduced migration of HCC cells through regulation of EMT. We constructed marvelD3-silenced and marvelD3-overexpression HCC cells (Figure 3(a,b)). Our results showed that the protein levels of E-cadherin were downregulated significantly, while those of vimentin were upregulated significantly in marvelD3-silenced HCC cells (Figure 3(c)). On the contrary, when we over expressed marvelD3, the protein level of E-cadherin was increased, and the protein level of vimentin was decreased (Figure 3(d)). Consistent with the result of Western blot, IF showed reduced expression of E-cadherin and enhanced expression of vimentin in HCC cells after marvelD3 knockdown (Figure 3(e)), as well reduced expression of vimentin and enhanced expression of E-cadherin in marvelD3-expression HCC cells (Figure 3(f)).

**(a) and (b)** RT-PCR was performed to examine the marvelD3 mRNA level. **(c) and (d)** MarvelD3, E-cadherin and Vimentin expression were assayed by western blot. **(e)** Immunofluorescence images show that marvelD3-knockdown downregulated the expression of E-cadherin and upregulated the expression of Vimentin (scale bar, 100 μm). **(f)** Immunofluorescence images show that marvelD3-overexpression upregulated the expression of E-cadherin and downregulated the expression of Vimentin (scale bar, 200 μm). (*P < 0.05, **P < 0.01)

### Effects of marvelD3 on HCC cell migration in vitro

**Figure 4.**
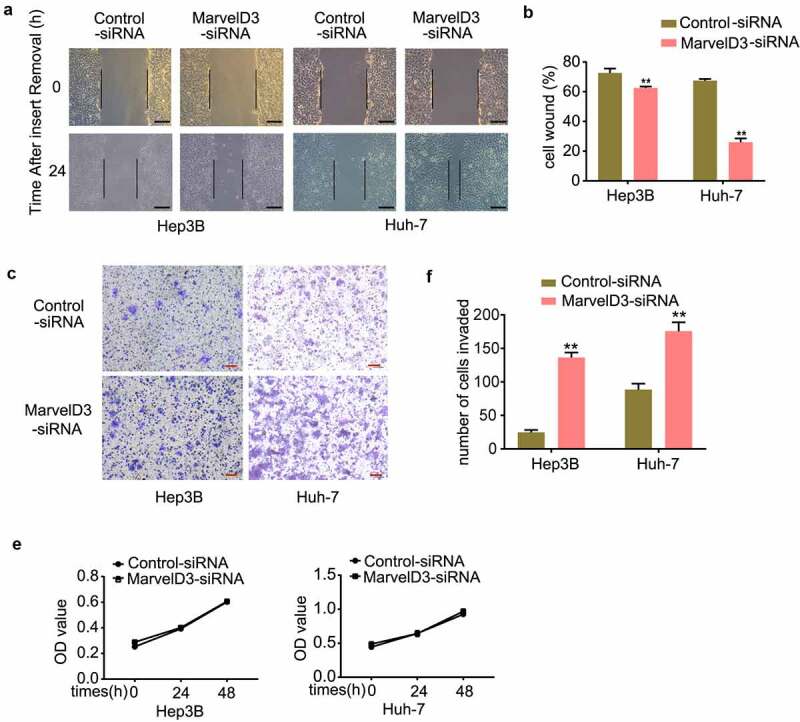
Effects of marvelD3 knockdown on HCC cell migration in vitro

**Figure 5.**
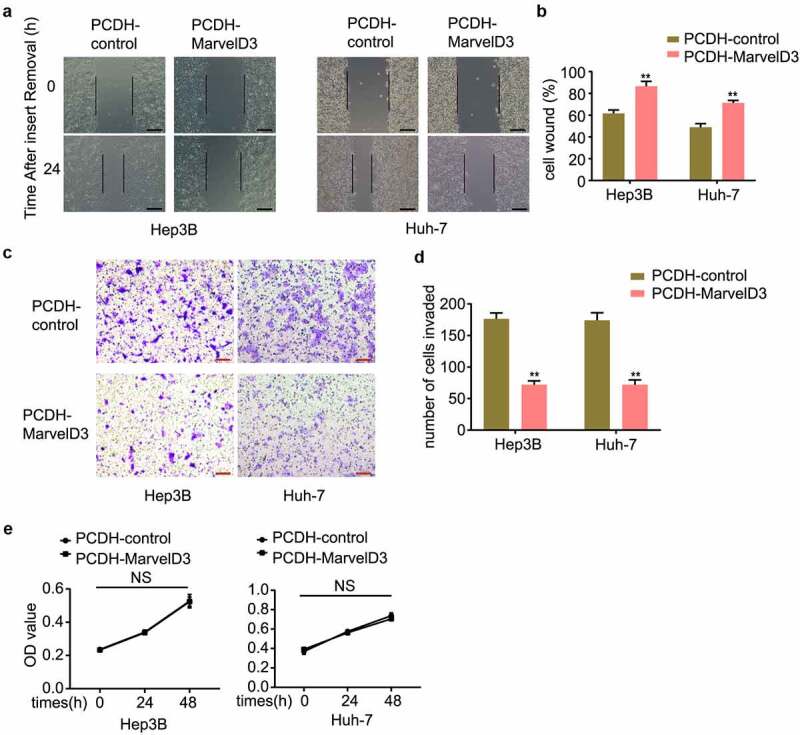
Effects of marvelD3 expression on HCC cell migration in vitro

EMT endows cells with migratory and invasive properties [28], thus we detected the effects of MarvelD3 expression on inhibition of HCC cell migration *in vitro*. The migratory effect of marvelD3 on HCC cells was assessed by wound healing and transwell migration assays. In marvelD3-silenced HCC cells, tumor cell Wound healing and their Transwell migration capacity were upregulated significantly (Figure 4(a-d)). However, marvelD3 knockdown did not affect tumor cell proliferation *in vitro* (Figure 4(e)). In marvelD3 overexpression HCC cells, tumor cell Wound healing and their Transwell migration capacity were down-regulated significantly, and tumor cell proliferation didn’t change obviously (Figure 5(a-e)). These results demonstrated that marvelD3 reduced HCC cell migration.

**(a)** Wound healing assay was used to detect the wound healing capacity in HCC cells treated with or without marvelD3 knockdown (100×). **(b)** The bar chart showed healing ratio. **(c) and (d)** Representative images from the transwell migration assay in marvelD3-silenced HCC cells (scale bar, 100 μm). Data represent the mean±SD. *P < 0.05, **P < 0.01. **(e)** The proliferation capacities of marvelD3-silenced HCC cells were detected by MTS.

**(a)** Wound healing assay was used to detect the wound healing capacity in marvelD3 expression HCC cells (100×). **(b)** The bar chart showed healing ratio. **(c) and (d)** Representative images from the transwell migration assay in marvelD3 expression HCC cells (scale bar, 100 μm). Data represent the mean±SD. *P < 0.05, **P < 0.01. **(e)** The proliferation capacities of marvelD3 expression HCC cells were detected by MTS.

### Effects of marvelD3 on inactivation of the NF-kB signaling pathway

**Figure 6.**
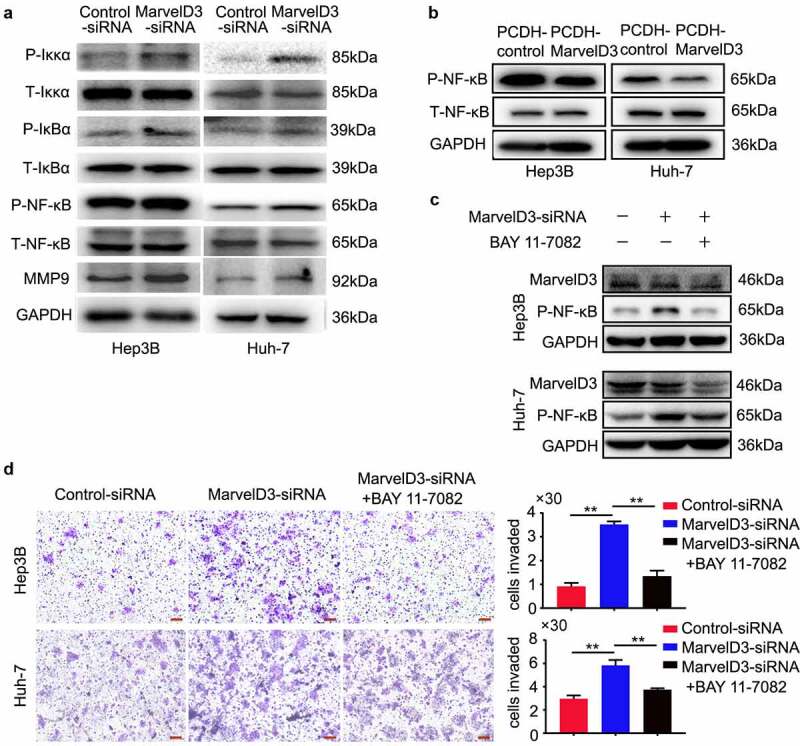
Effects of marvelD3 on inactivation of the NF-kB signaling pathway

NF-κB, a master regulator of EMT and cell metastasis, may be a target for prevention or treatment of HCC [29,30]. TJs suppresses invasion and metastasis of cells through NF-κB pathway [25]. Translocation of NF-κB-p65 into the nucleus and phosphorylation of IKK-β and IκBα are markers of NF-κB pathway activation. Our western blot data revealed NF-κB pathway activation in marvelD3-silenced HCC cells (Figure 6(a)). We also analyzed the effect of marvelD3 on expression of NF-κB downstream genes such as MMP9 and found that marvelD3 knockdown upregulated the expression of MMP9 (Figure 6(a)). And the expression of marvelD3 reduced the NF-κB pathway activation (Figure 6(b)). We next used NF-κB inhibitor BAY 11–7082 to block activity of NF-κB in marvelD3-silenced HCC cells (Figure 6(c)). As a result, we observed reduced migration of marvelD3-silenced HCC cells treated with NF-κB inhibitor BAY 11–7082 (Figure 6(d)). These data demonstrated that marvelD3 inhibited HCC cells migration partly through the NF-κB pathway.

**(a)** NF-kB pathway proteins including Ikkα, IkBα, NF-kB and MMP9 were measured by western blot in HCC cells where marvelD3 was knocked down. **(b)** The activity of NF-kB were detected by western blot in marvelD3 expression HCC cells **(c)** NF-kB pathway inihibitor BAY 11–7082 was applied to marvelD3-silenced HCC cells. Western blot was used to verify the expression of NF-kB. **(d)** Representative images from the transwell migration assay demonstrated the migration capacity in marvelD3-silenced HCC cells treated with NF-kB pathway inihibitor BAY 11–7082, with bar charts showing migration numbers (right panel). Magnification, 100× . Scale, 100 μm. (*P < 0.05, **P < 0.01).

## Discussion

In this study, we found that marvelD3 was downregulated in HCC tissues and its downregulation was obviously correlated with the tumor stage. Additionally, our result suggested that TGFβ-1 stimulation and snail or slug transfection reduced expression of marvelD3. The change of marvelD3 effected EMT markers and migration of HCC cells. Mechanistic analysis suggested that marvelD3 inhibited HCC progression by inactivating the NF-κB pathway.

As a kind of tight junction (TJ)-associated protein, claudins are useful molecular markers for various cancers [31–33]. A recent study has demonstrated that TJ protein expression is altered in HCC and cholangiocarcinoma [34]. For example, claudin-1 has been shown to be upregulated in advanced liver disease and HCC, and differential claudin-4 expression facilitates distinguishing between these two forms of cancer at the molecular level. Another report also revealed that claudin-3 inhibits cancer aggressiveness and is a potential prognostic biomarker for HCC [9]. However, in the MARVEL family, occludin, tricellulin, and marvelD3, little is known about changes in their expression during cancer progression. MarvelD3 has been reported to play a significant inhibitor role in colon adenocarcinoma cells and pancreatic cancer cells [6,12]. In this study, we found that tumor tissue had lower expression level of marvelD3 than normal tissue and expression of marvelD3 was associated with TNM stage of HCC. These results indicate that marvelD3 plays a role as a tumor suppressor gene in tumorigenesis.

EMT is a multistep biological process whereby epithelial cells change by transient dedifferentiation to a mesenchymal phenotype. During carcinoma progression, EMT plays a crucial role in the early steps of metastasis when cells lose cell-cell contacts by ablation of E-cadherin. Strong inducers of EMT such as TGF-β orchestrate both fibrogenesis and carcinogenesis. Additionally, the SNAI family with its members Snail (SNA1) and Slug (SNAI2) is involved in the activation of EMT. Snail is the prominent inducer of EMT in HCC [35]. In our study, we specifically examined changes in marvelD3 during TGF-β-induced EMT and overexpression of snail and slug in HCC cells. With downregulation of E-cadherin and upregulation of vimentin, expression of marvelD3 was decreased. A previous study has suggested that, under hypoxia and treatment with TGF-β1, claudin-1 is decreased [6], and claudin-3 knockdown affects regulation of EMT gene expression in vitro [20]. Several other studies have shown that marvelD3 expression is more strongly reduced in cell lines with invasive phenotypes derived from breast, pancreatic, and prostate tumors than relatively normal cell lines [12]. Similar to previous studies, we established marvelD3-silenced and marvelD3 overexpression HCC cells. Then we observed a significant change in EMT markers. These results indicate that marvelD3 plays an important role in the process of EMT and the further research of the correlation with marvelD3 and EMT may be valuable. The main characteristic of EMT is promotion of cell migration. Our data demonstrated that marvelD3 was indeed involved in EMT-related processes. Therefore, we speculated that marvelD3 inhibited migration of HCC cells. For verification, we compared the migratory capacities of marvelD3-silenced and normal HCC cells. As a result, we observed a stronger migration capacity in marvelD3-silenced HCC cells compared with the normal group, while expression of marvelD3 reduced the migration capacity of HCC cells. This result suggests that marvelD3 inhibits migration of HCC cells.

To further explore the mechanism of marvelD3 in the inhibition of EMT, we also carried out an in-depth analysis of cell signaling pathway. The cross-talk between TGF-β signaling and NF-κB is critical for various biological processes, including the development of different liver diseases [22,36,37]. Furthermore, accumulating studies had demonstrated that NF-kB signaling pathway is essential for the induction and maintenance of EMT in a large number of cancers [30]. The NF-κB signaling pathway stimulus to the regulation of hepatic fibrosis and HCC cell is recognized by receptors and then transmitted into the cell. This process is achieved through adaptor signaling proteins activating IKK. IKK phosphorylates IκB in the cytoplasm, which leads to degradation of IκB and release of NF-κB from the inhibitory complex. Subsequently, NF-κB protein translocates into nucleus where it binds to target sequences and activates gene transcription [38]. In our study, change of marvelD3 regulated phosphorylation of IKK-β and IκBα and nuclear translocation of p65, which suggested that knockdown of marvelD3 activated the NF-κB pathway and further promoted expression of NF-κB downstream target gene MMP9. To verify this result, NF-κB activity inhibitor BAY 11–7082 was used to treat cells and the result demonstrated that inhibition of marvelD3 in HCC cell migration was partly mediated through the NF-κB pathway. However, it is a limitation that we had not further investigated other effective signal pathways, which could be involved in the effect of marvelD3 on HCC progress.

In summary, our study showed that expression of marvelD3 was downregulated in human HCC tissues and HCC cells with TGF-β1/snail-induced EMT. In vitro experiments revealed that marvelD3 inhibited EMT and invasion of HCC cells via suppression of the NF-κB signaling pathway. Therefore, marvelD3 may be an effective inhibitor of EMT and migration in human HCC cells. This suggests that marvelD3 can be further evaluated as a novel biomarker to predict the prognosis of HCC and as a therapeutic target for of HCC.

## Supplementary Material

Supplemental Material

## Data Availability

All data generated or analyzed during this study are basically included in this published article (and its supplementary information files).
