# Supplementary material for: Role of tight junction-associated MARVEL protein marvelD3 in migration and epithelial–mesenchymal transition of hepatocellular carcinoma
Source: Cell Adh Migr. 2021 Aug 2;15(1):249–60. doi: 10.1080/19336918.2021.1958441 (PMC8331009; doi:10.1080/19336918.2021.1958441)
Supplement: Supplemental Material [file KCAM_A_1958441_SM2224.zip › suppl/Supplementary Materials Table S1.docx]

Table S1. Demographics of HCC patients.

| tumor tissue |
| --- |
| normal liver tissue |
| tumor and adjacent non-tumor |

| Pathological diagnosis | Sex | Age | TNM tumor stage  (TnN0M0) | Application |
| --- | --- | --- | --- | --- |
|  | Male | 49 | T3 | IHC |
|  | Male | 58 | T1 | IHC |
|  | Male | 64 | T2 | IHC |
|  | Male | 56 | T2 | IHC |
|  | Male | 63 | T2 | IHC |
|  | Male | 51 | T3 | IHC |
|  | Male | 60 | T3 | IHC |
|  | Male | 50 | T2 | IHC |
|  | Male | 56 | T1 | IHC |
|  | Male | 63 | T2 | IHC |
|  | Male | 62 | T3 | IHC |
|  | Male | 58 | T2 | IHC |
|  | Male | 52 | T2 | IHC |
|  | Male | 61 | T2 | IHC |
|  | Male | 63 | T3 | IHC |
|  | Male | 45 | T1 | IHC |
|  | Male | 49 | T3 | IHC |
|  | Male | 52 | T1/adjacent non-tumor | IHC |
|  | Male | 51 | T3/adjacent non-tumor | IHC |
|  | Male | 51 | T2/adjacent non-tumor | IHC |
|  | Male | 51 | T1/adjacent non-tumor | IHC |
|  | Male | 27 | T3/adjacent non-tumor | IHC |
|  | Male | 55 | T2/adjacent non-tumor | IHC |
|  | Male | 45 | T3/adjacent non-tumor | IHC |
|  | Male | 36 | T1/adjacent non-tumor | IHC |
|  | Male | 59 | normal liver tissue | IHC |
|  | Male | 40 | normal liver tissue | IHC |
|  | Male | 63 | normal liver tissue | IHC |
|  | Male | 57 | normal liver tissue | IHC |
|  | Male | 54 | normal liver tissue | IHC |
|  | Male | 61 | T3 | WB/PCR |
|  | Male | 37 | T2 | WB/PCR |
|  | Male | 56 | T1 | WB/PCR |
|  | Male | 44 | T1 | WB |
