# Supplementary material for: Role of tight junction-associated MARVEL protein marvelD3 in migration and epithelial–mesenchymal transition of hepatocellular carcinoma
Source: Cell Adh Migr. 2021 Aug 2;15(1):249–60. doi: 10.1080/19336918.2021.1958441 (PMC8331009; doi:10.1080/19336918.2021.1958441)
Supplement: Supplemental Material [file KCAM_A_1958441_SM2224.zip › suppl/Supplementary Materials Table S2.docx]

Table S2. q-RT-PCR primers for *MarvelD3, E-cadherin, Vimentin* and *GAPDH*

| Gene | Forward primer | Reverse primer |
| --- | --- | --- |
| *MarvelD3-V1* | GAACCCCCTTCGGAGAGATA | CGGCAAGGACAAAGTAGGAG |
| *MarvelD3-V2* | TTACCAGTCAGAGGCGGAAG | CCCCCTGTGGAACTGTAAGA |
| *E-cadherin* | CGAGAGCTACACGTTCACGG | GGGTGTCGAGGGAAAAATAGG |
| *Vimentin* | AGTCCACTGAGTACCGGAGAC | CATTTCACGCATCTGGCGTTC |
| *GAPDH* | GAGTCAACGGATTTGGTCGT | GACAAGCTTCCCGTTCTCAG |
